# Touch events and human activities for continuous authentication via smartphone

**DOI:** 10.1038/s41598-023-36780-3

**Published:** 2023-06-29

**Authors:** Vincenzo Gattulli, Donato Impedovo, Giuseppe Pirlo, Francesco Volpe

**Affiliations:** Dipartimento di Informatica, Università degli studi di Bari Aldo Moro, 70125 Bari, Italy

**Keywords:** Computer science, Scientific data

## Abstract

The security of modern smartphones is related to the combination of *Continuous Authentication* approaches, *Touch events, and Human Activities*. The approaches of *Continuous Authentication, Touch Events, and Human Activities* are silent to the user but are a great source of data for Machine Learning Algorithms. This work aims to develop a method for continuous authentication while the user is sitting and scrolling documents on the smartphone. Touch Events and Smartphone Sensor Features (from the well-known *H-MOG Dataset*) were used with the addition, for each sensor, of the feature called *Signal Vector Magnitude.* Several *Machine Learning Models* have been considered with different experiment setups, *1-class,* and *2-class,* for evaluation. The results show that the 1-class SVM achieves an accuracy of 98.9% and an F1-score of 99.4%, considering the selected features and the feature *Signal Vector Magnitude* very significant.

## Introduction

Protecting smartphones is one of the main challenges in cybersecurity. *Knowledge-based approaches* are authentication methods that verify user identity based on secret mnemonic knowledge. Human tends to want to memorize simple information and passwords, being simple and short can easily be guessed and stolen. In addition, the peculiarity of *Knowledge-Based approaches* is that they are one-shot: authentication is performed only once and is no longer required while using the smartphone. In fact, after performing the first and unique authentication, an attacker, since that moment, impersonates the victim. These actions can then fall into personal violence or cyberbullying^1^.

Scientific research is moving toward implementing approaches called Continuous Authentication and approaches that combine *Touch Events and Human Activities*^2^. Continuous Authentication tends to perform more of a security check while the user uses the device. Touch and Human Activities approach, as with Continuous Authentication approaches, depend on characterizing the user's behavior while using, in this case, the smartphone. The behavior identifies the user because they naturally touch the screen like no other user and walk or run like no other user. Touch Events (*moments when the user touches the screen*) and Human Activities (*Walking, Running, Jumping, Fall, etc....)*^3^ are intrinsic to user behavior and can, together, identify them. In addition, continuous authentication approaches have the advantage of working in the background (*silently to the user*). This latter aspect increases the usability of the approach because it becomes universal without adding additional hardware or requiring specific actions to be performed by the user.

This work is focused on a specific approach that ties into Continuous Authentication and Touch and Human Activities events from smartphones. A method is developed for continuous user authentication while using a smartphone by identifying possible illegitimate users during sitting and reading activity (*scrolling a document in the background on the smartphone screen*). Touch event-related features and sensor-related features, including accelerometer, magnetometer, and gyroscope, were considered in this experiment. For each sensor (X, Y, Z), the Signal Vector Magnitude was considered, thus producing four features (X, Y, Z, M). In addition, experiments are performed on a portion of the HMOG dataset that, as mentioned earlier, characterizes the action of sitting while scrolling through a chat/document. Machine learning models trained with GENUINE and IMPOSTOR features with two different setups (1-class and 2-class) are used to evaluate the experiment. The result is valid as a comparison between the selected machine learning models to decree and tests the selected features if they perform for the document reading and sitting task.

To the best of the authors’ knowledge, this is the very first experiment in this direction, that is, to consider this inherent combination of Touch Events and Human Activities with a portion of the H-MOG dataset inherent in the activity of reading a document and being seated via smartphone.

The structure of this paper is as follows: Chapter 2 analyzes the current relevant works in this field. In Chapter 3, the materials used for the study are presented. The methodology employed is described in Chapter 4. Chapter 5 outlines the experimental setup employed in this research. The performance of the models derived from the experiments is evaluated in Chapter 6. Finally, in Chapter 7, the conclusions drawn from this study are presented.

## Related works

Biometrics is grouped into two categories: behavioral biometrics and physiological biometrics. Physiological biometrics is based on a person's physical attributes such as fingerprints, finger or palm veins, face shape, DNA, handprint, hand geometry, iris, or eye retina recognition. On the other hand, behavioral biometrics is closely related to a person's habits, such as typing rhythm, gait, and voice. Behavioral biometrics enables continuous and passive authentication. This means behavioral characteristics are continuously captured and compared with the user's profile throughout the session, not just at log-in. Behavior profiling is considered in many studies.

Numerous studies deal with the problem of continuous authentication using the accelerometer, magnetometer, and gyroscope as sensors. In Zhu et al.^4^, a framework, SenSec, is presented that constantly collects sensory data from accelerometers, gyroscopes, and magnetometers and builds the gesture model of how a user uses the device. *SenSec* calculates the confidence that the mobile device is being used by its owner. The authors show that this framework can achieve 75% accuracy in identifying users and 71.3% accuracy in identifying non-owners, with only 13.1% false alarms. In Lee et al.^5^, researchers design a system based on multiple sensors that continuously learn the owner's behavior patterns and the characteristics of the environment and then authenticates the current user without interrupting user-smartphone interactions. This method can adaptively update the user's model by considering the temporal change of the user's patterns. Experimental results show that the method provides more than 90% accuracy. The method also shows that the combination of multiple sensors provides better accuracy. In Amini et al.^6^, motion sensors embedded in available smartphones are utilized to learn users' behavioral characteristics during interaction with the mobile device and provide an implicit re-authentication mechanism. This approach uses time and frequency domain features extracted from motion sensors and a short-term memory model (LSTM) with negative sampling to build a re-authentication framework. The framework can re-authenticate a user with 96.70% accuracy in 20. In Ehatisham-ul-Haq et al.^7^ authentication framework is proposed that provides a platform for multi-class user authentication using twelve extracted features. It is reported that the Bayes Net classifier provides the best performance for activity recognition on the device regarding EER accuracy and computation time required for activity classification. In Abuhamad et al.^8^, AUToSen, a deep-learning-based active authentication approach, is proposed, demonstrating that AUToSen works accurately using readings from only the three sensors. The use of one-second sensor data allows an F1 authentication score of approximately 98%, a false acceptance rate (FAR) of 0.95%, a false rejection rate (FRR) of 6.67%, and an equal error rate (EER) of 0.41%. In Mekruksavanich et al.^9^, a new continuous authentication framework called DeepAuthen is introduced. It identifies smartphone users based on their smartphones' physical activity patterns measured by the accelerometer, gyroscope, and magnetometer sensors. Scientists conduct a series of tests on user authentication using different deep learning classifiers and a new deep learning network called DeepConvLSTM.

Some studies need to consider the use of the three sensors. Some researchers use only accelerometer data as a sensor. In particular, Kwapisz et al.^10^ researchers collect accelerometer data from thirty-six users while performing normal daily activities such as walking, jogging, and climbing stairs. They then aggregate these time series data and apply classification algorithms to the resulting data to generate predictive models. In Centeno et al.^11^, an approach based on a deep learning autoencoder is studied that achieves an EER of 2.2% in real-world scenarios. The system uses accelerometer data. In addition, the sensing process is carried out in the cloud to reduce the computational load of the smartphone.

Another approach is proposed by Li et al.^12^, in which only two sensors are used. Researchers present SCANet, a continuous authentication system based on two-stream convolutional neural networks that use the accelerometer and gyroscope of smartphones to monitor users' behavioral patterns. The system uses the two-stream CNN to learn and extract representative features. With the features extracted from the CNN, SCANet uses the class support vector machine to train the classifier in the enrolment phase. The experimental results show that the CNN achieves 90.04% accuracy, and SCANet achieves an average of 5.14% equal errors.

However, not all studies on passive and continuous authentication are based on using sensors in smartphones. Some research in the literature studies the problem of using the touchscreen to detect a legitimate user. For instance, Frank et al.^13^ propose 30 haptic data features obtained from users interacting with a smartphone by performing basic navigation operations such as up-down and left-right scrolling. The trained classifier obtained an EER of 0% for intra-session authentication, 2–3% for between-session authentication, and less than 4% when the authentication test was performed one week after the registration phase. Garbuz et al.^14^ present a continuous user authentication system based on user interaction with the touchscreen in combination with micro-movements performed simultaneously by smartphones. Two of the users’ most common gestures (vertical swipes up and down and taps) are considered. The researchers use the One-Class Support Vector Machine algorithm to obtain a model of a legitimate user. The results show that the legitimate user is blocked on average after 115-116 gestures (*a combination of swipes and taps*), and an imposter is detected in 2-3 gestures. Shen et al.^15^ considered four common types of touch operations, features are extracted to characterize users' touch behavior, and one-class classification algorithms are used. The results are a FAR of 4.68% and an FRR of 1.17%.

Other studies combine several approaches to study the problem. For example, some researchers use sensors and touchscreen data. Volaka et al.^16^ examines the impact of using the touchscreen and sensor-based features in an authentication model using deep learning methods. A three-level deep neural network is constructed on the combined feature sets. The results achieved 88% accuracy and EER values of 15%. In Incel et al.^17^, researchers examine whether it can continuously authenticate users via behavioral biometrics on a mobile banking application. A continuous authentication scheme, called DAKOTA is developed that records data from the phone's touch screen and motion sensors to monitor and model the user's behavioral patterns. The results reveal that binary-SVM has an EER of 3.5 percent. Another approach is considered in Smith–Creasey et al.^18^, where facial and haptic modalities are combined, demonstrating that a stacked classifier can improve continuous authentication on mobile devices. An EER of 3.77% for a single sample is achieved.

Data security in the way of smartphones is critical. Defining a secure device goes through standard or continuous authentication and *general security issues, Data Analysis, Energy Efficiency, and Anomalous Behavior*. In security, issues such as edge computing are highly relevant in smartphone authentication. Edge computing could provide greater security and reduce latency while performing authentication^19^. Device security also passes through device immunity it is essential to protect it from possible data poisoning performed by third parties^20^. Smartphone protection also involves data analysis to optimize smartphone processes and identify likely suspicious patterns^21^. In addition to processes, energy efficiency is essential in smartphones, so optimizing energy would lead to improved security in authentication and user experience with the same^22^. Finally, the anomaly can also be identified by people's abnormal behavior (Smart City scope), a concept emphasized in this paper, through analysis of smartphone data generated^23^.

## Material

In order to extract features considering Touch Events and Human Activities from smartphones, the H-MOG dataset (A Multimodal Data Set for Evaluating Continuous Authentication Performance)^24^ has been used. The dataset has three user usage scenarios or activities: Reading Documents, Text Writing, and Navigating a Map to locate a Destination.

The dataset has been built adopting an Android smartphone to record the data stream related to the Touch and Hardware Sensors installed in the device in real time. This was performed to capture user behavior. One hundred users were recruited to experiment. Users are randomly assigned a session to read, write or navigate the map. A session lasts about 5–15 min, and each user has 24 sessions (eight reading sessions, eight writing sessions, and eight map navigation sessions). Each user contributes about 2–6 h of behavioral traits.

The collected data are stored in CSV files. Data acquisition from the sensors has a sampling rate of 100 Hz. Nine categories of data are collected^24^:

1. *Accelerometer* Timestamp, Acceleration along X/Y/Z-Axis.
2. *Gyroscope* Timestamp, Rotation Rate along X/Y/Z-axis.
3. *Magnetometer* Timestamp, Ambient Magnetic Field along X/Y/Z-axis.
4. *Raw touch event* timestamp, finger count, finger ID, raw touch type, X/Y coordinate, contact size, screen orientation.
5. *Tap gesture* timestamp, tap type, raw touch type, X/Y coordinate, contact size, screen orientation.
6. *Scale gesture* timestamp, pinch type, time delta, X/Y focus, X/Y span, scale factor, screen orientation.
7. *Scroll gesture* starting and current timestamp, X/Y coordinate, and contact size; speed along X/Y coordinate; screen orientation.
8. *Fling gesture* starting and ending timestamp, X/Y coordinate, and contact size; speed along X/Y coordinate; screen orientation.
9. *Keypress on virtual keyboard* timestamp, press type, key ID, screen orientation.

## Method

This chapter describes the adopted approach using the following Pipeline: Dataset and pre-processing, features extraction, models, and evaluation.

In this case, the design pipeline is more concerned with the *Dataset* and the *Features Extracted* for model evaluation. The first phase (A) contemplates the information about the sample extracted from the H-MOG Dataset and the importance of cleaning the dataset from incorrect detections, which affects more the preprocessing phase. The second phase (B) involves the extraction of features from the raw data of the dataset. Phase (B) prepares the data for the machine learning models that are mentioned later and the methods for evaluating them (Phase (C)).

### Dataset and pre-processing

Twenty users were considered in this experiment, including those who performed the *"Reading Documents"* usage scenario. The activity is reading a document from a smartphone while strolling through it with the finger (*Touch Event*) and while sitting (*Human Activity*). The hardware sensor of the device picks up the accelerometer, gyroscope, and magnetometer triaxial.

**Figure 1:**
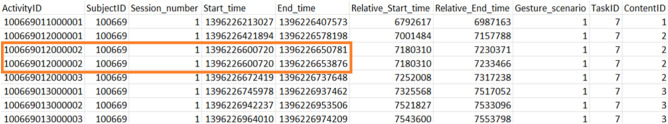
For activity 100,669,012,000,002 two records have the same activity start time and a different activity end time.

From a preliminary analysis of the dataset, repeated activities are identified. Some activities have the same code, and the same activity starts time but with a different end time. Since it is impossible to understand why this situation occurs, removing the records related to these activities is preferred. An example is shown in Fig. 1:

The dataset is preprocessed to identify reading session 1 among the 20 selected users. The session activities are different for each. For example, in the first session, one user might have performed a writing activity, while another might have performed a map browsing activity.

### Feature extraction

In this work, the following sensors are considered for each user: Event Touch for sensors accelerometer, magnetometer, and gyroscope. Each sensor contains, among other data, the X, Y, and Z coordinates. These data are augmented with the *“Signal Vector Magnitude”* calculated on each sensor as follows:

$$M= \sqrt{{X}^{2}+{Y}^{2}+{Z}^{2}}$$

Concerning the *Event Touch*, each of these events has a system time that indicates the moment when the user makes a touch on the screen. For each *Event Touch*, was considered the following time interval:

$$ \left[ {SYSTIME \, - \, 100{\text{ms}}, \, SYSTIME \, + \, 100{\text{ms}}} \right] $$

**Figure 2:**
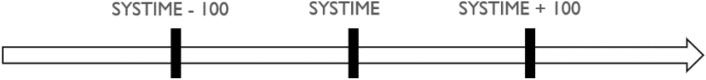
Time points.

This time interval is used to extract features from the sensor. For each sensor coordinate, the sensor data's maximum, minimum, mean, and standard deviation lie between SYSTIME-100 ms and SYSTIME, between SYSTIME and SYSTIME + 100 ms, and the difference of the values 100 ms before and between the values 100 ms after are calculated. An explanatory image of the time points is shown in Fig. 2.

For example, is considered the X direction of the accelerometer:

- The *MAXIMUM, MINIMUM, AVERAGE*, and *DVST* of the X data in the interval between [*systime-100, systime*];
- The *MAXIMUM, MINIMUM, AVERAGE*, and *DVST* of the X data in the interval between [*systime, systime* + *100*];
- The differences between the values 100 ms before and 100 ms after.

Performing a calculation inherent to the feature calculated in this study: four sensors axis (X, Y, Z, M), three sensors (*Accelerometer, Gyroscope, Magnetometer*), and 12 features extracted from each sensor, there are 144 features (4*3*12). Moreover, seven following additional features are added:

1. *Gesture_scenario*
2. *Task_id*
3. *Pointer_count*
4. *Pointer_id*
5. *Action_id*
6. *Content_id*
7. *Phone_orientation*

In total, for each user, there are 151 features.

### Model and evaluation

The classification problem has been considered in two different approaches. In the first case, a binary classification has been performed between the target (authorized) users’ class and the non-authorized (impostor) class. The following Machine Learning Models are adopted: Decision Tree, Random Forest, and Multi-layer perceptron. Regarding the second case, the 1-class SVM classifier was considered. These machine learning models were chosen because they are most used in the context of continuous authentication. Evaluation of the models is done by accuracy and f1-score, these metrics are among the most widely used for supervised approaches and are very significant metrics for data observation and analysis.

## Experiment setup

The first experiment considers 2-classes for each user: GENUINE and IMPOSTOR. The records of the GENUINE class identify the genuine user, and the records of the IMPOSTOR class are considered malicious users. More specifically, the IMPOSTOR class for each genuine user includes the entire set of features of the remaining 19 other users. The dataset is divided into Training and Test, respectively, 70–30% randomly. Finally, the average accuracy and f1-score of the twenty users are shown in the results table of the first experiment. An example of the first experiment is shown in Table 1.

**Table 1:** Table depicting GENUINE and IMPOSTOR users for modeling User 1.

User	Number of records	CLASS
Authorized user U1	1000	GENUINE
U3	995	IMPOSTOR
U4	993	IMPOSTOR
U5	1010	IMPOSTOR
U6	1015	IMPOSTOR
U7	1017	IMPOSTOR
U8	2300	IMPOSTOR
U9	2700	IMPOSTOR
…	…	…
U20	2200	IMPOSTOR

The second experiment considers 1-class to consider real cases in which impostors are not available in advance at training time. In fact, in the previous case, the assumption that the impostors are known at training time is very unreal. In this experiment, for each genuine user, a model is trained considering only its own 151 features, at testing time, the model is tested on the features of each of the 19 other users considered as never seen impostors. This process is carried out for each User of the twenty selected. Finally, the averages inherent in model accuracy and f1-score across all twenty users are extracted.

## Results

Table 2 shows results related to the first experiment. This Experimental setup confirms that both classes were correctly recognized in the test. The model that performed best was the Random Forest.

**Table 2:** Average results 20 users in 2-class.

	Accuracy	F1-score
Decision tree	0.95	0.93
Random forest	**0.96**	**0.95**
MLP	0.92	0.91

Significant values are in bold.

The result of the second experiment considers a more compliant and balanced model, in which each model is trained on the individual user and tested on each of the others. The average accuracy obtained is 98.9% (Table 3).

**Table 3:** Average results 20 users in 1-class.

#User	Accuracy	F1 score macro
Average	0.98	0.99

## Conclusions

The purpose of this work has been to develop a method for continuous user verification while using a smartphone and to identify illegitimate users during a reading activity (an activity that an illegitimate user, after stealing the smartphone device, could perform by reading and scrolling through a chat while comfortably sitting in a chair).

The set of raw features acquired by the sensors has been augmented by calculating the "Signal Vector Magnitude" feature. The classification problem has been considered a two-class problem and a one-class one. In the former case, the hypothesis is that impostor trials are available at training time, in the latter, the (real) hypothesis is that impostors are not known at training time. Regarding the models considered, the setup of the first experiment (2-class) decreed the Random Forest as the best model, while in the second setup test, the 1-class SVM performed well. Even if the results are encouraging, these conclusions cannot be generalized due to the limited number of users within the dataset.

As a future development, other bullying-related activities could be identified, and their authentication verified using smartphones. It is also essential to build an extended dataset so that more complex methods can be applied: Multi-speed transformer network^25^ e AUCO Resnet^26^. Another relevant point could be developing these solutions on a smartphone device.

## Data Availability

The datasets generated and/or analyzed during the current study are available in the Yang, Q. et al.^17^ repositories at https://hmog-dataset.github.io/hmog/. The Grant of License is reported in the previous link.
